# Estrogen Receptor Status in Relation to Risk of Contralateral Breast Cancer–A Population-Based Cohort Study

**DOI:** 10.1371/journal.pone.0046535

**Published:** 2012-10-08

**Authors:** Maria E. C. Sandberg, Per Hall, Mikael Hartman, Anna L. V. Johansson, Sandra Eloranta, Alexander Ploner, Kamila Czene

**Affiliations:** 1 Department of Medical Epidemiology and Biostatistics, Karolinska Institutet, Stockholm, Sweden; 2 Saw Swee Hock School of Public Health, Singapore, Singapore; 3 Department of Surgery, National University of Singapore, Singapore, Singapore; Wayne State University School of Medicine, United States of America

## Abstract

**Background:**

It is unclear whether estrogen receptor (ER)-status of first primary breast cancer is associated with risk of metachronous (non-simultaneous) contralateral breast cancer (CBC), and to what extent endocrine therapy affects this association.

**Methods:**

We studied the effect of ER-status of the first cancer on the risk of CBC overall, and for different ER-subtypes of CBC, using a large, population-based cohort. The cohort consisted of all women diagnosed with breast cancer in the Stockholm region 1976–2005; 25715 patients, of whom 940 suffered CBC. The relative risk was analyzed mainly using standardized incidence ratios (SIR).

**Results:**

Women with breast cancer had a doubled risk of CBC compared to the risk of breast cancer in the general female population (SIR: 2.22 [2.08–2.36]), for women with a previous ER-positive cancer: SIR = 2.30 (95% CI:2.11–2.50) and for women with a previous ER-negative cancer: SIR = 2.17 (95% CI:1.82–2.55). The relative risk of ER-positive and ER-negative CBC was very similar for women with ER-positive first cancer (SIR = 2.02 [95%CI: 1.80–2.27] and SIR = 1.89 [95%CI: 1.46–2.41] respectively) while for patients with ER-negative first cancer the relative risk was significantly different (SIR = 1.27 [95% CI:0.94–1.68] for ER-positive CBC and SIR = 4.96 [95%CI:3.67–6.56] for ER-negative CBC). Patients with ER-positive first cancer who received hormone therapy still had a significantly higher risk of CBC than the risk of breast cancer for the general female population (SIR = 1.74 [95% CI:1.47–2.03]).

**Conclusion:**

The risk of CBC for a breast cancer patient is increased to about two-fold, compared to the risk of breast cancer in the general female population. This excess risk decreases, but does not disappear, with adjuvant endocrine therapy. Patients with ER-positive first cancers have an increased risk for CBC of both ER subtypes, while patients with ER-negative first cancer have a specifically increased risk of ER-negative CBC.

## Introduction

Of all women with breast cancer, each year 0.6–0.7% will develop contralateral breast cancer (CBC) [1], [2], [3], [4], [5], [6], translating into approximately 10–15% of all breast cancer patients being diagnosed with CBC during the first 20 years after initial diagnosis [4], [7]. The risk of CBC does not seem to decline with time since first diagnosis [5], [8], it is however higher for patients that were young at their first breast cancer [9], [10], [11]. Further, CBC-patients have a considerably worse prognosis than patients with unilateral breast cancer, as the authors of this paper have shown previously [12].

Estrogen receptor (ER) status acts both as a prognosticator, independent of treatment, but also as a predictor of endocrine therapy response [13], [14], [15]. It is still not clear how hormone receptor status of the first breast cancer affects the risk of CBC. Several studies show that, among breast cancer patients there is no effect of ER-status of the first cancer on the risk of CBC-[16], [17], [18], [19], [20]. This is however not uncontroversial; two studies show that breast cancer patients with positive ER-status have lower risk of CBC compared to patients with ER-negative first breast cancer [21], [22]. Though it should be noted that neither of these two studies had the opportunity to account for endocrine therapy, one of the studies found this association to be effect modified by age at diagnosis and only apparent for young women [22]. Randomized trials have shown that adjuvant endocrine therapy decreases the risk of CBC overall [9], but there have been indications that endocrine therapy might increase the risk of ER-negative CBC substantially [23]. Many of the previous studies on this subject [16], [17], [18], [19], [20], [22] have been limited by small sample size (number of CBC cases ranging from 43 to 131) and/or have focused on only one aspect (i.e. only ER-status or only endocrine therapy) in the interplay between risk, ER-status of the two tumors and the treatment given. This study, on the other hand, aims at a comprehensive analysis of the relationship between ER-status of the first tumor, endocrine therapy and ER-status of the second tumor and has a sufficient sample size (N = 695) to confidently answer these questions. We conducted a population-based analysis, contrasting the risk to develop CBC among breast cancer patients to the risk to develop breast cancer among the general female population.

## Methods

### Ethics statement

This study was approved by the ethical committee at the Karolinska Institutet, Stockholm, Sweden. As the study was purely register based, no contact was made with the study persons and the data were analyzed anonymously informed consent was not obtained. The exception from informed consent was confirmed by the ethical committee.

### Study population

The Stockholm Breast Cancer Register is a population-based register to which all breast cancer patients in the Stockholm-Gotland health-care region of Sweden are reported. The register is complete from 1976 and contains, among other clinical variables, date of birth, date of diagnosis, ER-status and information on subsequent cancers, distant metastasis and date of death. Adjuvant therapy is included in the register from 1990. In this study we included all women who were recorded in the Stockholm Breast Cancer Register during 1976–2005 as having invasive breast cancer occurring as the patient's first malignancy and not diagnosed in TNM-stage 4 (N = 25 715). We investigated the risk of CBC, defined as invasive contralateral breast cancer occurring as the patient's second malignancy and not diagnosed in TNM-stage 4. By these restrictions we tried to ensure that the breast cancers were primary malignancies, and not misclassified metastatic events.

Further, women with CBC that occurred less than 3 months after the first breast cancer could not be part of the study population, since we regard their two cancers as simultaneous. The cutoff of 3 months for simultaneous CBC has been used previously by our group and others [24], [25], [26]. Consequently, while the population at risk of CBC is breast cancer patients, the population at risk for simultaneous CBC is breast-cancer free women, i.e. the general female population. Furthermore, in our risk calculations we differentiate between the first and second cancer, which is not possible among simultaneously occurring CBCs.

The measurement of ER-status during the study period is naturally of crucial importance for this study. Prior to 1988 isoelectric focusing [27] was used to assess ER-status, from 1988–2003 an ELISA assay (Abbott Laboratories kit) [28] was used, and during the two last years of this study immunohistochemistry (DAKO Laboratories kit) was used. The measurements from these methods have been shown to be highly correlated [29], [30] and also to correlate well with more recent methods, like RT-PCR [31]. The ELISA assay measured fmole/µg DNA and any tumor with a concentration of ER ≥0.05 fmol/µg DNA was defined as ER-positive, tumors measured with the immunohistochemistry method were classified as positive if 10% or more of the tumor cells expressed ER.

For the analysis of endocrine therapy we restricted our cohort to patients with their first cancer diagnosed after 1990 (approximately 60% of the cohort), since treatment information was not included in the register until then. Endocrine treatment was recorded as yes/no, and for one third of the patients the type of endocrine therapy was also specified. (Tamoxifen for 75% of the patients and aromatase inhibitors for 22%.)

### Statistical analysis

Incidence of CBC was estimated as the number of cases divided by total person time (person-years) at risk, from first diagnosis of breast cancer until date of CBC or censoring (date of malignancy at any other site, date of distant metastasis, date of death or December, 31, 2005, whichever came first). The cumulative incidence of CBC was estimated by the Nelson-Aalen estimator [32] and is shown graphically for CBC overall, ER-positive CBC and ER-negative CBC, by ER-status of the first cancer. The log-rank test with 5% significance level was used to assess whether the cumulative incidence differed between patients with ER-positive and ER-negative first cancer.

We used the standardized incidence ratio (SIR) to investigate the risk of CBC among breast cancer patients, compared to the risk of unilateral breast cancer in the general (breast-cancer free) female population. We calculated the ratio of observed to expected number of CBC-cases, in 5-year age intervals and 5-year calendar-period intervals. The background rate used for calculating the expected number of CBCs was the 5-year-age- and 5-year-period-specific rate of breast cancer in Stockholm during the study period (1976–2005), which we calculated from population counts of the general population of women and number of unilateral breast cancer patients in the Stockholm Breast Cancer Register. We calculated the rates separately for all breast cancer, for ER-positive cancer and for ER-negative cancer, thereby enabling us to compare the rate of e.g. ER-negative CBC to the rate of ER-negative unilateral breast cancer. We used 95% confidence intervals (CI) based on the Poisson distribution for the SIR to determine statistical significance [33].

As a sensitivity analysis we also calculated the SIRs restricted to patients with known ER-status (also of the second cancer, if present), with the aim of investigating the potential effect of missingness of ER-status. Further, we investigated whether the risk of CBC, as compared to the risk of breast cancer in the general population, differed depending on how long time had passed since the first breast cancer. In effect, the observed over expected ratio of CBC during the first five years from diagnosis of primary cancer was calculated, by counting the accumulated person time (which, by using the background rate for the general population, gave rise to the expected number of CBCs) and observed number of CBCs, during these first five years. Then, the number of observed CBCs occurring more than 5 years after the first breast cancer and the person time accumulated from the time point five years after first primary diagnosis and until the end of follow up was calculated, giving rise to an observed over expected ratio in this group. These two ratios could then be compared and whether they differ will answer the question of whether the risk of CBC, as compared to the risk of breast cancer in the general population, differ by time since the first breast cancer.

All data preparation and analyses were done using SAS Statistical Package 9.2.

## Results

The final cohort consisted of 25 715 breast cancer patients, of which 18 853 (73%) had known ER-status; 14 720 had ER-positive breast cancer and 4 133 had ER-negative cancer (Table 1). Among these 25 715 women 940 contralateral breast cancers were diagnosed; 553 with ER-positive first cancer, 142 with ER-negative and 245 with unknown ER-status at first cancer. The mean age at (first) breast cancer diagnosis was 61.1 years (range: 18–101) and the mean age at CBC was 63.6 years (range 23–90). The overall incidence of CBC was 454 cases per 100 000 person-years (PYR), the specific incidence of ER-positive and ER-negative CBC was 219 and 78 per 100 000 PYR, respectively. The overall 5-year incidence decreased from 533 cases per 100 000 PYR, for women diagnosed with their first cancer in 1976–1980 to 322 cases per 100 000 PYR for women diagnosed with their first cancer in 1996–2000. Among the patients diagnosed with (first) breast cancer after 1990 (N = 15 145) 69% received endocrine therapy, 24% received no endocrine therapy and endocrine therapy was unknown for 7% of the patients. 349 CBC-cases arose in this restricted cohort.

**Table 1:** Characteristics of the unilateral breast cancer cohort and the CBC-cohort; 1976–2005 in the Stockholm-Gotland region.

	Breast cancer*	CBC
**No of patients**	25 715	940
**Age at (first) diagnosis**		
<45	3059 (12%)	152 (16%)
45–55	5728 (22%)	275 (29%)
55–65	6445 (25%)	244 (26%)
65–75	5606 (22%)	202 (21%)
>75	4877 (19%)	67 (7%)
**Calendar period of (first) diagnosis**		
1976–1985	6426 (25%)	375 (40%)
1985–1995	8900 (35%)	430 (46%)
1995–2005	10 389 (40%)	135 (14%)
**Time between first and second cancer**		
Mean years (std dev)	-	6.9 (5.4)
Estrogen receptor status:		
**Positive at first cancer**	14 720 (57%)	553 (59%)
Positive at second cancer		292 (31%)
Negative at second cancer		66 (8%)
Unknown at second cancer		195 (21%)
**Negative at first cancer**	4133 (16%)	142 (15%)
Positive at second cancer		50 (5%)
Negative at second cancer		49 (3%)
Unknown at second cancer		43 (5%)
**Unknown at first cancer**	6862 (27%)	245 (26%)
Positive at second cancer		111 (12%)
Negative at second cancer		46 (5%)
Unknown at second cancer		88 (9%)

* This cohort is the study population for this study, it includes all breast cancer patients, both those that will develop CBC and those that will not.

CBC = contralateral breast cancer. Std dev = standard deviation.

**Figure 1:**
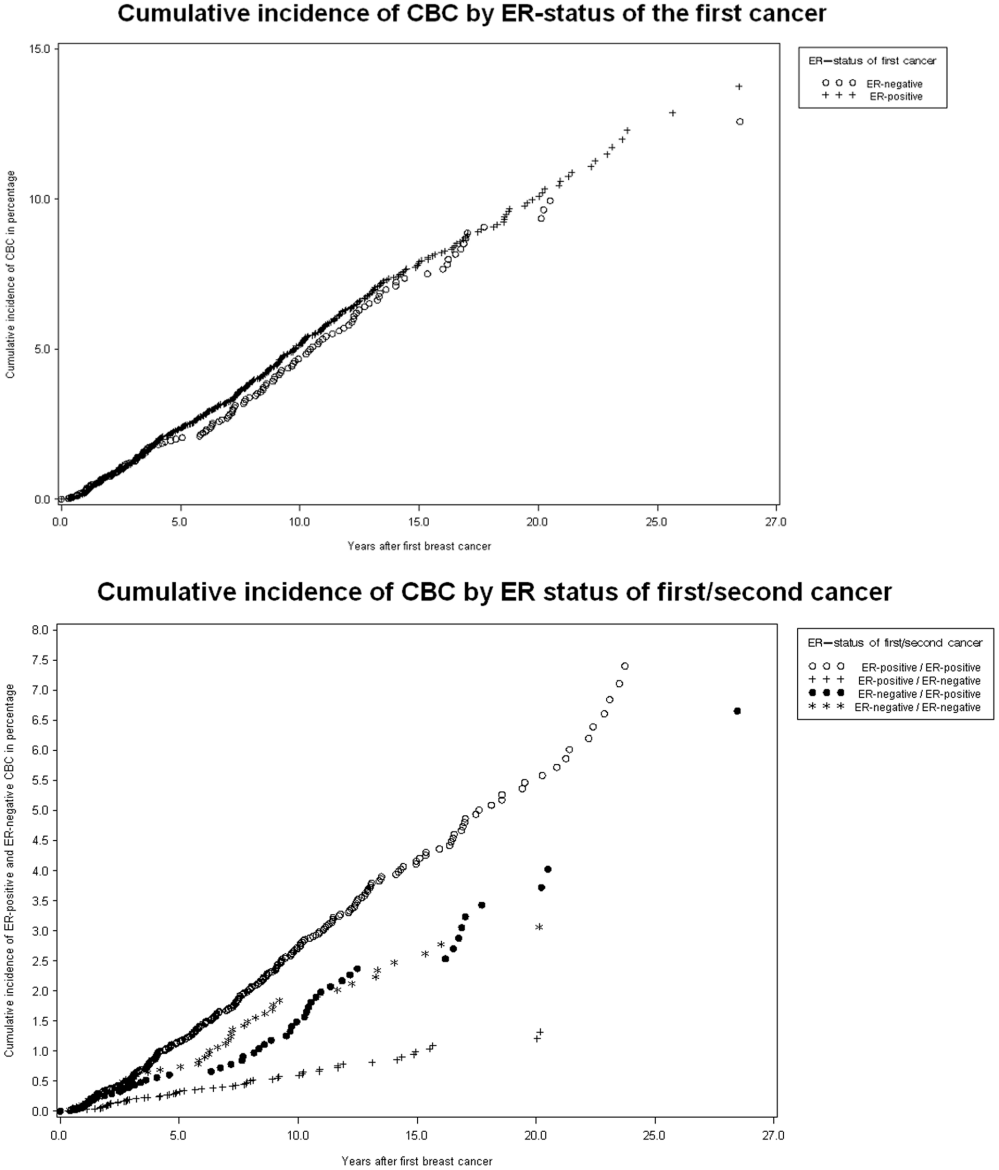
Nelson –Aalen plots. Cumulative incidence of CBC for women with ER-negative vs. ER-positive first breast cancer. Panel A shows the cumulative incidence of CBC of any ER-subtype by ER-status of the first cancer. The p-value for the log-rank test of cumulative incidence of CBC overall was 0.338, thus the cumulative incidence of CBC did not differ between patients with ER-positive vs. ER-negative first primary breast cancer. Panel B shows the cumulative incidence of ER-positive and ER-negative CBC by ER-status of the first cancer. (The p-value for the log-rank test of cumulative incidence of ER-positive CBC show significant difference between patients with ER-positive vs. ER-negative first breast cancer (P-value = <0.001) and similarly the p-value for the log-rank test of cumulative incidence of ER-negative CBC also show significant difference between patients with ER-positive vs. ER-negative first breast cancer (P-value = <0.001). See Table 5 for number of women at risk.

The cumulative incidence of CBC given the ER-status of the first cancer is shown by Nelson-Aalen plots in Figure 1. The incidence of CBC overall is given in panel A and panel B shows the incidence of ER-positive and ER-negative CBC by ER-status of the first cancer. The cumulative incidence of CBC overall was not statistically different depending on ER-status for the first cancer (P = 0.338), but the cumulative incidence of ER-positive CBC was statistically significantly higher for patients with ER-positive first cancer, compared to patients with ER-negative first cancer (P<0.001), and likewise was the cumulative incidence of ER-negative CBC statistically significantly higher for patients with ER-negative first cancer, compared to patients with ER-positive first cancer (P<0.001). These analyses was based only on the patients for which ER-status (also of the second cancer, if present) is known (N = 18 615). This cohort was used also in panel A, where information of ER-status of the second cancer is not strictly needed, to enable comparison between the panels.

The risk for breast cancer patients to develop CBC was 2.22 times higher (95% CI: 2.08–2.36) than the risk of the general female population to develop unilateral breast cancer (Table 2). Further, we found a SIR of 2.30 (95% CI: 2.11–2.50) when separately investigating the risk of CBC among patients with ER-positive first breast cancer, the corresponding relative risk among patients with ER-negative first cancer was 2.17 (95% CI: 1.82–2.55). Overall, the relative risk of CBC is significantly higher for the younger patients (i.e. women diagnosed with their first breast cancer before the age of 50), the risk is two to three-fold higher than for patients diagnosed with their first cancer at/after the age of 50 (Table 2). When investigating the SIRs closer (<5 years) and further (>5 years) from first diagnosis we did not find any evidence of a difference between these two relative risks (data not shown).

**Table 2:** Standardized incidence ratios (SIR) comparing the incidence of CBC to the incidence of unilateral breast cancer, overall and according to ER-status of the first breast cancer, for all patients and stratified on age.

			Risk of CBC		
	Women at risk N	Person years at risk	Observed cases *N*	SIR	95% CI
**Overall**					
All first breast cancer	24 775	192 247	940	**2.22**	**2.08–2.36**
ER-positive first breast cancer	14 167	108 298	553	**2.30**	**2.11–2.50**
ER-negative first breast cancers	3 991	30 573	142	**2.17**	**1.82–2.55**
**Patients diagnosed before age of 50**					
All first breast cancer	5 357	23 927	152	**5.73**	**4.86–6.72**
ER-positive first breast cancer	2 822	11 783	79	**5.87**	**4.64–7.31**
ER-negative first breast cancers	1 190	5 397	29	**5.15**	**3.45–7.40**
**Patients diagnosed at/after age of 50**					
All first breast cancer	19 418	168 320	788	**1.98**	**1.85–2.13**
ER-positive first breast cancer	11 345	96 515	474	**2.08**	**1.90–2.28**
ER-negative first breast cancers	2 801	25 176	113	**1.89**	**1.55–2.26**

26% of the CBC-cases had unknown ER-status at their first cancer, 34% of the CBC-cases had unknown ER-status of their second cancer.

SIRs standardized for age at diagnosis in 5-year-categories and for period of diagnosis in 5-year-categories.

SIR = Standardized incidence ratio, CI = Confidence Interval, CBC = contralateral breast cancer, ER = Estrogen receptor.

As a sensitivity analysis we restricted our cohort to only the patients for which ER-status (also of the second cancer, if present) was known (N = 18 615), the purpose was to investigate the effect of missingness of ER-status on our estimates. We calculated the risk for breast cancer patients to develop CBC compared to the risk of the general female population to develop unilateral breast cancer; SIR = 2.02 (95% CI = 1.84–2.21, N = 457). The relative risk among women with ER-positive first cancer was 2.02 (95% CI = 1.81–2.24, N = 358) and among women with ER-negative first cancer 2.03 (95% CI = 1.65–2.47, N = 99). These findings are thus in concordance with the risk estimates for the full cohort (Table 2). In addition, we also conducted a sensitivity analysis of the definition of non-simultaneous CBC; using a cutoff of one year between the first and second cancer, instead of three months. The results were however very similar (see supplementary online material, Table S1).

When investigating the relative risk of ER-positive and ER-negative CBC separately (Table 3) we found that the risk of CBC compared to unilateral breast cancer is statistically significantly increased in all ER-subgroups except for ER-positive CBC among the patients with ER-negative first cancer. Further, we found no significant difference between the relative risk of ER-positive and ER-negative CBC for patients with ER-positive first cancer (SIR = 2.02 [95%CI: 1.80–2.27] and SIR = 1.89 [95%CI: 1.46–2.41] respectively). In contrast, for patients with ER-negative first cancer the risk was notably and statistically significantly different; patients with ER-negative first cancer had no increased risk of ER-positive CBC (SIR = 1.27 [95% CI: 0.94–1.68]) but an almost five-fold increased risk for ER-negative CBC (SIR = 4.96 [95%CI: 3.67–6.56]). The risks for both ER-positive and ER-negative CBC was further increased among women younger than 50 years at first diagnosis, most notably for ER-negative CBC (Table 3).

**Table 3:** Standardized incidence ratios (SIR) comparing the incidence of ER-positive and ER-negative CBC to the incidence of unilateral breast cancer, overall and according to ER-status of the first breast cancer, for all patients and stratified on age.

	Risk of ER-positive CBC			Risk of ER-negative CBC			P-value of Wald test
	Observed cases *N*	SIR	95% CI	Observed cases *N*	SIR	95% CI	
**Overall**							
All first breast cancer	453	**1.78**	**1.62–1.96**	161	**2.60**	**2.21–3.03**	**<0.01**
ER-positive first breast cancer	292	**2.02**	**1.80–2.27**	66	**1.89**	**1.46–2.41**	**0.63**
ER-negative first breast cancers	50	1.27	0.94–1.68	49	**4.96**	**3.67–6.56**	**<0.01**
**Patients diagnosed before age of 50**							
All first breast cancer	54	**3.61**	**2.71–4.71**	37	**7.02**	**5.94–9.67**	**<0.01**
ER-positive first breast cancer	38	**4.97**	**3.52–6.81**	8	**3.00**	**1.29–5-91**	**0.19**
ER-negative first breast cancers	5	1.58	0.51–3.70	15	**13.20**	**7.39–21.77**	**<0.01**
**Patients diagnosed at/after age of 50**							
All first breast cancer	399	**1.66**	**1.51–1.84**	124	**2.19**	**1.82–2.61**	**<0.01**
ER-positive first breast cancer	254	**1.86**	**1.64–2.10**	58	**1.80**	**1.37–2.33**	**0.82**
ER-negative first breast cancers	45	1.25	0.91–1.67	34	**3.89**	**2.69–5.44**	**<0.01**

26% of the CBC-cases had unknown ER-status at their first cancer, 34% of the CBC-cases had unknown ER-status of their second cancer.

SIRs standardized for age at diagnosis in 5-year-categories and for period diagnosis in 5-year-categories.

SIR = Standardized incidence ratio, CI = Confidence Interval, CBC = contralateral breast cancer, ER = Estrogen receptor.

We evaluated the effect of endocrine therapy for the first cancer for women with ER-positive breast cancer by calculating the SIRs for the patients for whom we had information on endocrine therapy (patients diagnosed from 1990). The overall relative risk of CBC, as well as the risk of ER-positive CBC, was lower for the endocrine-therapy treated women (SIR for CBC overall = 1.74 (95% [CI: 1.47–2.03]), compared to non-endocrine treated (SIR for CBC overall = 2.81 [95% CI: 1.98–3.87]), while no corresponding risk reduction was found for ER-negative CBC (Table 4).

**Table 4:** Standardized incidence ratios (SIR) comparing the incidence of CBC to the incidence of unilateral breast cancer, for CBC overall and for specific ER-status of first and second breast cancer, taking hormone therapy for the first cancer into account.

	Women at risk *N*	Person years at risk	Observed cases *N*	Expected cases *N*	SIR	95% CI
**All CBC** *						
Hormone therapy treated ER-positive first cancer	7711	38 365	156	89.86	**1.74**	**1.47–2.03**
Non-hormone therapy treated ER-positive first cancer	835	6 214	37	13.15	**2.81**	**1.98–3.87**
**ER-positive CBC**						
Hormone therapy treated ER-positive first cancer	7711	38 365	87	56.88	**1.53**	**1.23–1.87**
Non-hormone therapy treated ER-positive first cancer	835	6 214	22	7.26	**2.80**	**1.75–4.23**
**ER-negative CBC**						
Hormone therapy treated ER-positive first cancer	7711	38 365	26	13.03	**2.00**	**1.30–2.92**
Non-hormone therapy treated ER-positive first cancer	835	6 214	3	2.00	1.51	0.31–4.41

* ER-status of the second cancer was unknown for 29% of the CBC-patients.

The cohort is restricted to patients diagnosed after 1990, since hormone therapy is not recorded in the register before then. SIR calculations were stratified on hormone therapy treatment for first breast cancer and adjusted for age at first breast cancer in 5-year-categories and for period of first breast cancer in 10-year-categories.

SIR = Standardized incidence rate, CI = Confidence Interval, CBC = contralateral breast cancer, ER = Estrogen receptor.

**Table 5:** Number of women at risk of CBC by year after diagnosis, subdivided on ER-status of the first cancer.

Number of women at risk					
ER-status of first cancer	Year of follow-up				
	0	5	10	15	20
ER-positive	14 525	3 436	2 280	1 222	901
ER-negative	4 090	783	535	364	358

## Discussion

We found the risk of CBC for breast cancer patients to be about twice as high compared to the risk of breast cancer in the general (breast-cancer free) female population, independently of ER-status of the first cancer. However, while ER-positive breast cancer seems to predict an increased risk for CBC in general, an ER-negative first cancer seems to specifically increase the risk for ER-negative CBC, and not for ER-positive CBC. The risk of CBC is highly increased for all women with first breast cancer diagnosed before the age of 50, and we now show that this increase is more pronounced for ER-negative CBC. Endocrine therapy for the first cancer decreases the risk of CBC, compared to when no endocrine therapy is given, this decrease was mediated through the risk of ER-positive CBC; no effect is seen for the risk of ER-negative CBC.

Strengths of this study include that it was conducted in a country with a unified healthcare system and a population-based cancer register, which enabled close to complete case identification. We reduced the risk of misclassification of CBC through exclusion of women diagnosed with a previous cancer other than breast cancer, women diagnosed with an initial TNM stage IV breast cancer and women diagnosed with distant metastasis prior to the second cancer, this strict definition is a potential explanation for the somewhat lower incidence (454 cases per 100 000 person years) shown in this study, compared to other studies [1], [2], [3], [4], [5], [6], [7]. Among the limitations is the lack information on HER2-status, since it was not been measured during the majority of the study period. Further, due to a high degree of missing information in the register we chose to not include progesterone receptor (PR) status in the analysis. However, considering the stronger endocrine therapy response prediction conferred by ER-status and the high concordance between PR-status and ER-status we believe that the loss information by not including PR-status is not significant. Further, misclassification of ER-status cannot be excluded since the measurements were preformed with different methods. However, this misclassification of ER in relation outcome status, if at all present, ought to be non-differential (as there is no reason to believe that the misclassification of ER-assessment of the second cancer would be dependent on the ER-status of the first cancer) and would as such bias our results towards the null.

We found breast cancer patients to have approximately doubled risk of CBC, compared to the risk of breast cancer in the general female population, and found no significant difference in relative risk of CBC by ER-status of the first cancer (Table 2). ER-status of the first cancer can thus not be used clinically for predicting the risk of CBC (Table 2 and Figure 1A). However, the relative risk of the two ER subtypes of CBC is quite different depending on the ER-status of the first cancer; the risk of ER-positive CBC is higher if the first cancer is ER-positive, and the risk for ER-negative CBC is substantially increased if the first cancer was ER-negative (Table 3 and Figure 1B). This may however primarily be seen as a sign of the previously shown high concordance of ER-status in CBC tumors [12], [17], [34], [35] and is not currently of clinical importance.

The findings in Table 3 indicate that an ER-positive first breast cancer is a marker of increased risk of CBC in general, while an ER-negative first breast cancer specifically increases the risk for ER-negative CBC. This might imply that the underlying host factors (environmental or genetic) causing ER-negative breast cancer are relatively stronger compared to the host factors causing ER-positive breast cancer. Despite extensive studies of potential host factors (primarily genetic factors) increasing the risk of ER-negative breast cancer specifically, while being less important for ER-positive breast cancer, only one such factor of significance has been found; BRCA-1 [36] (but there are indications that certain single nucleotide polymorphisms are more important for ER-negative breast cancer, this was recently shown also for CBC [37]). On the other hand, several life style risk factors e.g; nulliparity and late childbearing have been shown to increase the risk of ER-positive breast cancer but do not seem to affect the risk of ER-negative breast cancer [38], [39], [40]. Unknown risk factors could thus be the reason for the higher incidence of ER-negative CBC following ER-negative first cancer, we believe that there is a need for studies elucidating this.

The two previous studies that have investigated the SIR of CBC using unilateral breast cancer as the background population have conflicting results. Bouchardy et al. [41] showed, in a small study of 63 CBC-patients, no increased risk of CBC overall among breast cancer patients compared to the risk of breast cancer in the general female population. This finding is rather unexpected, in contrast to the well-established high risk of yet another breast cancer for breast cancer patients, and thereby potentially puts into question the validity of the results. Kurian et al [21] assessed hormone receptor status rather than ER and progesterone receptor (PR) separately; hormone receptor-positive was defined as ER- and/or PR-positive. They showed, in consistency with our results, that patients with hormone receptor-positive first breast cancer have approximately twice the risk of CBC compared to the risk of breast cancer in the general female population, and the relative risk increase was independent of ER-status of the second cancer. However, Kurian et al. also showed a more than three-fold risk of CBC if the first cancer was ER-negative, which was driven by the almost 10 times increased relative risk of ER-negative CBC for patients with ER-negative first cancer. Kurian et al found that women who were young at diagnosis and women belonging to ethnic groups other than non-Hispanic whites had the highest relative risks of ER-negative CBC following an ER-negative first cancer, this could potentially explain the discrepancy between that study and ours, since both these groups are significantly more frequent in the study by Kurian et al.

As previously shown [42], [43], [44] we note that the incidence of CBC is higher among patients diagnosed with the first cancer at an early age; the overall risk increase for breast cancer patients compared to the general female population is two-fold, but for women diagnosed before the age of 50 it is close to 6-fold (IRR: 5.73[95% CI: 4.86–6.72]) (Table 2). In contrast to the previous studies of the effect of age at first diagnosis, we had the possibility to separate the effect between ER-positive and ER-negative cancer (Table 3). The effect seems to be more apparent for the ER-negative cancers; in the young cohort the risk of ER-negative CBC is more increased than the risk of ER-positive CBC. A tentative explanation for this finding would be that both ER-negativity and young age at diagnosis implies a relatively stronger genetic (compared to the environmental) influence on the risk of breast cancer, that these two factors coincide further increase the probability of a genetic makeup rendering the woman susceptible for (yet another) breast cancer.

We found the incidence of CBC overall to decrease over calendar period, as both our group and others have shown previously [45], [46], a finding that is most likely due to the widespread use of adjuvant chemo- and endocrine therapy in the later decades, which markedly decrease the risk of CBC [9]. While there, for chemotherapy, exists neither epidemiological evidence nor any biological mechanism to suggest that the effect would be different for ER-positive and ER-negative cancers, this is not the case for endocrine therapy, which we therefore investigated further. The effect size of endocrine therapy in our study is in the same order as the effect size shown in a meta analysis of randomized clinical trials (SIR for treated: 1.74 [95% CI: 1.47–2.03] vs. SIR for untreated: 2.81 [95% CI: 1.98–3.87]) [9]. The risk decrease is further enhanced when investigating the risk for ER-positive CBC and not present for ER-negative CBC, which is in accordance with the biological mechanism for endocrine therapy. In contrast to the findings by Li et al [23], [47], we found no statistically significant increase of the risk of ER-negative CBC for women treated, compared to not treated, by endocrine therapy, but due to low number of ER-negative CBC among non-treated women further confirmation might be needed.

In conclusion; the increased risk of CBC for breast cancer patients compared to the risk of breast cancer for the general female population is not dependent on ER-status of the first cancer, which can thus not be used for risk prediction. However, it seems that ER-positive breast cancer predicts an increased risk for CBC in general while an ER-negative first cancer specifically increases the risk for ER-negative CBC. This, in combination with our finding that the increased risk compared to the general population is further enhanced for young women and in particular for ER-negative CBC, might imply effects of host factors that are yet unknown. Finally, the protective effect of adjuvant endocrine therapy for the first breast cancer is exclusively seen for the risk of ER-positive CBC, however also these treated women have a significantly higher risk of CBC than the risk of breast cancer for general female population.

## Supporting Information

Table S1 **SIR sensitivity analysis latency time.** Standardized incidence ratio (SIR) comparing the incidence of CBC to the incidence of unilateral breast cancer, overall and according to ER-status of the first and second breast cancer, including only CBCs with one year or more between the two cancers.
(DOCX)
