# Supplementary material for: Estrogen Receptor Status in Relation to Risk of Contralateral Breast Cancer–A Population-Based Cohort Study
Source: PLoS One. 2012 Oct 8;7(10):e46535. doi: 10.1371/journal.pone.0046535 (PMC3466301; doi:10.1371/journal.pone.0046535)
Supplement: Table S1 — SIR sensitivity analysis latency time. Standardized incidence ratio (SIR) comparing the incidence of CBC to the incidence of unilateral breast cancer, overall and according to ER-status of the first and second breast cancer, including only CBCs with one year or more between the two cancers. (DOCX) [file pone.0046535.s001.docx]

## Standardized incidence ratio (SIR) comparing the incidence of CBC to the incidence of unilateral breast cancer, overall and according to ER-status of the first and second breast cancer

|  |  |  | **Risk of CBC** | | |
| --- | --- | --- | --- | --- | --- |
|  | Women at risk N | Person years at risk | Observed cases *N* | SIR | 95% CI |
| **Overall** |  |  |  |  |  |
| All first breast cancer | 24 775 | 192 247 | 940 | **2.22** | **2.08 – 2.36** |
| ER-positive first breast cancer | 14 167 | 108 298 | 553 | **2.30** | **2.11 – 2.50** |
| ER-negative first breast cancers | 3 991 | 30 573 | 142 | **2.17** | **1.82 – 2.55** |
| **Sensitivity analysis*** |  |  |  |  |  |
| All first breast cancer | 24 775 | 173 290 | 884 | **2.30** | **2.15-2.46** |
| ER-positive first breast cancer | 14 167 | 97 588 | 435 | **2.41** | **2.20-2.62** |
| ER-negative first breast cancers | 3 991 | 27 418 | 132 | **2.21** | **1.85-2.62** |

|  | **Risk of ER-positive CBC** | | | **Risk of ER-negative CBC** | |  |
| --- | --- | --- | --- | --- | --- | --- |
|  | Observed  cases *N* | SIR | 95% CI | Observed cases *N* | SIR | 95% CI |
| **Overall** | | | |  | | |
| All first breast cancer | 453 | **1.78** | **1.62 – 1.96** | 161 | **2.60** | **2.21 – 3.03** |
| ER-positive first breast cancer | 292 | **2.02** | **1.80 – 2.27** | 66 | **1.89** | **1.46 – 2.41** |
| ER-negative first breast cancers | 50 | 1.27 | 0.94 – 1.68 | 49 | **4.96** | **3.67 – 6.56** |
| **Sensitivity analysis*** | | | |  | | |
| All first breast cancer | 435 | **1.90** | **1.73-2.09** | 149 | **2.68** | **2.27 - 3.15** |
| ER-positive first breast cancer | 282 | **2.18** | **1.94-2.45** | 63 | **2.02** | **1.56 - 2.59** |
| ER-negative first breast cancers | 47 | 1.32 | 0.97-1.76 | 45 | **5.07** | **3.70 - 6.79** |

SIRs standardized for age at diagnosis in 5-year-categories and for period diagnosis in 10-year-categories.

SIR = Standardized incidence ratio, CI= Confidence Interval, CBC= contralateral breast cancer, ER=Estrogen receptor.

*Sensitivity analysis= cutoff between synchronous (not included in analysis) and metachronous was 1 year, instead of three months
